# Risk of Venous Thromboembolism in Infectious Diseases: A Literature Review

**DOI:** 10.3390/pathogens14080816

**Published:** 2025-08-18

**Authors:** Ilaria Pati, Francesca Masiello, Vanessa Piccinini, Lucia De Fulvio, Maria Simona Massari, Vincenzo De Angelis, Mario Cruciani

**Affiliations:** National Blood Centre, Italian National Institute of Health, 00161 Rome, Italy; francesca.masiello@iss.it (F.M.); vanessa.piccinini@iss.it (V.P.); mariasimona.massari@guest.iss.it (M.S.M.); vincenzo.deangelis@iss.it (V.D.A.); crucianimario@virgilio.it (M.C.)

**Keywords:** venous thromboembolism (VTE), risk of VTE, infectious diseases, COVID-19, HIV infection, tuberculosis, HCV infection, CMV infection, bacterial infections

## Abstract

Systemic or localized infections increase the risk of venous thromboembolism (VTE). All types of infection can elevate the risk of VTE thrombosis, although some appear to increase risk more than others. In the current narrative review, we seek to overview the available evidence related to the epidemiology of VTE caused by infections. We focused on patients with infection in community setting or hospitalized, on patients with COVID-19, HIV infection, tuberculosis, HCV infection, and CMV infection, as well as on individuals with other types of infection that might increase the risk of VTE. Moreover, we tried to evaluate how the risk of VTE in person with different types of infections could be addressed in clinical practice with the use of anticoagulants. Extended VTE prophylaxis may not be warranted for all infections, but may be very helpful for some, such as those with intra-abdominal infection, systemic bloodstream infection, lower respiratory infection, and symptomatic urinary tract infection.

## 1. Introduction

Venous thromboembolism (VTE), manifesting as deep venous thrombosis (DVT) and pulmonary embolism (PE), is an important cause of cardiovascular morbidity and mortality. In most cases PE occurs as a consequence of DVT; however, in some cases it can also occur without a previous diagnosis of DVT [1,2,3,4,5]. Approximately 10 million cases of VTE are reported to occur worldwide each year. The underlying pathophysiological mechanisms by which venous thrombi are formed consist of stasis of venous blood flow, hypercoagulability, and damage to the blood vessel wall [6]. In addition to the classic risk factors that predispose to VTE (prolonged immobilization, use of oral contraceptives, orthopedic surgery, cancer, spine injuries, and limb fractures), thrombosis is also a common consequence of infectious disease [4,7,8]. Acute infection and subsequent systemic inflammation may contribute to an increased risk of VTE. Systemic or localized infections increase the risk of thrombosis from 2 to 20 times and are independent risk factors for thromboembolic diseases such as DVT and PE as well as cardiovascular (myocardial infarction) and cerebrovascular events (stroke) [8,9]. The key difference between infection-associated thrombosis and thrombosis in other circumstances is a strong inflammatory response to the infectious agent, necessary for host defense, leading to the overexpression of inflammatory mediators. Transcription factors and intracellular enzymes are involved in inflammatory reactions, causing the secretion of various inflammatory mediators including cytokines and chemokines and growth factors, responsible for endothelial cell, leukocyte, and platelet activation; activation can determine damage to the endothelium, which ensures blood flow and vascular homeostasis, resulting in loss of its anticoagulant and anti-inflammatory properties, fibrin deposition, and thrombus formation [10,11]. All types of infection can elevate risk of thrombosis, although some appear to increase risk more than others. The most dramatic example of infection associated thrombosis is represented by the recent COVID-19 pandemic caused by SARS-CoV-2 [12,13,14]. The observed risk of VTE in COVID-19 is high (up to 30%), particularly in intensive care unit (ICU) patients. In these cases, the host immune response is the main trigger for vascular thromboembolic events during SARS-CoV-2 infection. The high number of patients with thrombotic complications suggests that the main cause of coagulopathy, with systemic vascular damage, is the direct action of the virus on the endothelium. In COVID-19 patients, the inflammatory state is an important trigger for the activation of the coagulation cascade. The activation of monocytes and endothelial cells can determine the expression of tissue factor with consequent thrombin genesis. Elevated levels of pro-inflammatory cytokines and chemokines (e.g., tumor necrosis factor (TNF)-α, interleukin (IL)-8, and IL-6) can inactivate natural anticoagulant pathways and activate steps of the coagulation cascade.

Other infections that are associated with a high risk of VTE are tuberculosis and human immunodeficiency virus (HIV) infection, but also hepatitis C virus (HCV) and cytomegalovirus (CMV) infections have been associated with increased risk of VTE [15,16,17,18]. Hospitalization with infection as a whole or of specific sites (e.g., respiratory tract infections, urinary tract infections, intrabdominal and bloodstream infections) are also common trigger of VTE [3,19,20]. Since thrombosis is observed after infection with a diverse 66 range of pathogens, it is conceivable that the risk of post-infection thrombosis is influenced not only by factors derived from the pathogen but also by the host [21].

In the current comprehensive narrative review, we seek to overview the available evidence related to the epidemiology of VTE caused by infections. For this purpose, we reviewed the evidence from case reports, case controls and cohort studies, review articles and systematic reviews. We focused on patients with infection, both in community and hospital settings, on patients with COVID-19, HIV infection, tuberculosis, and HCV infection as well as on individuals with other types of infections that might increase the risk of thrombosis. Moreover, we tried to evaluate how the risk of VTE in person with different types of infections could be addressed in clinical practice with the use of anticoagulants.

The bibliographic search was conducted on MEDLINE (through PubMed) from inception up to April 2025. The search comprised combination of the following keywords: “Venous thromboembolism, Thromboembolisms, Thrombosis, Pulmonary embolism” AND “infectious diseases, or SARS-CoV-2/COVID-19, HIV, Hepatitis C virus (HCV), Epstein Barr virus, Varicella zoster, Cytomegalovirus, Mycobacterium tuberculosis, pulmonary infections, sepsis”. Of the 3988 records identified, 3796 were excluded because they were not related to thromboembolism and infectious disease. After screening of title and abstract, the full texts of 192 reports were downloaded and examined (91 related to TE and COVID-19, 20 to TE and HIV, 17 to TE and infectious diseases, 8 to TE and tuberculosis, 32 to TE and other viral infections, 4 to respiratory infections and TE, and 20 to thromboprophylaxis and infectious diseases, mostly related to COVID-19).

## 2. Risk of VTE in Hospitalized Patients with Infection and in Community Setting

Data from randomized controlled clinical trials (RCTs), cohort studies, population-based controlled studies, and nationwide databases show that acute infections increase the risk of VTE, as summarized in Table A1 [2,3,9,19,20,22,23,24,25,26,27,28,29,30,31,32]. The risk of VTE is significantly increased in acute infections diagnosed in hospital or treated in the community [24]. VTE is a serious health problem, especially in hospitalized elderly people. There is an association between hospitalization with infection and subsequent short-term VTE risk that exceeds the known association between hospitalization and VTE [28].

Hospitalization with infection is a strong VTE trigger also in non-immobilized patients. Infection and immobilization had a synergistic effect on the VTE risk [20]. Results from a large community-based case–control study show that after adjusting for other common VTE risk factors, any infection (pneumonia, urinary tract infections (UTI), intrabdominal infections, blood stream infections) increased significantly the probability of VTE (OR 2.4; 95% CI: 1.8, 3.2; *p* < 0.0001) [19]. Respiratory infections have widely been associated with an increased risk of VTE. In medically ill patients, case–control studies revealed that patients with pneumonia have a 2.5–5-fold and 8-fold higher risk, respectively, of VTE and PE, compared to controls [20,23,28,32]. Although this risk is highest within the first 2 weeks of acute respiratory infection, it can persist for months after the infection has resolved [8]. Studies related to VTE risk among surgical patients demonstrated that the diagnosis of pneumonia was associated with a 2–3-fold increase in the rates of postsurgical VTE, and that the diagnosis of pneumonia was also shown to be an independent risk factor for VTE in surgical ICU patients [32,33]. Cumulative incidence of VTE among patients with pneumonia admitted to the ICU is equal to 7% [32]; furthermore, the risk of VTE was higher without thromboprophylaxis, and was reduced by 50% with thromboprophylaxis in the first 2 weeks of ICU hospitalization. This finding emphasizes the need for accurate identification of hospitalized patients at risk of VTE in order to provide effective prophylaxis.

Acute infections are associated with a transient increased risk of VTE events even in community setting [8,9,24,26]. Respiratory tract, urinary tract, skin, intra-abdominal, and bloodstream infections treated in the community were associated with a 2.6-fold (95% CI: 2.5–2.8) increase in VTE risk [24]. The risk of VTE is substantially increased within 90 days after community-acquired bacteraemia when compared to controls. However, since the risk of VTE in patients treated for bacterial infection in the community with outpatient parenteral antimicrobials (OPA) is low (≤0.5%), the routine application of VTE prophylaxis would not seem justified [26].

## 3. Risk of VTE in COVID-19 Patients

COVID-19 can lead to systemic coagulation activation and thrombotic complications. Hospitalized patients, especially in the case of subjects hospitalized in ICU, may encounter a cascade of coagulopathies, which may lead to macro vessel thrombotic events such as PE, DVT, or arterial thromboembolism (ATE) [12,13,14]. The rate of thromboembolic complications in COVID-19 patients is unquestionably high. A large number of clinical trials have been published starting from 2020 on this timely and relevant topic, and these trials have been the objects of more than one hundred systematic reviews and meta-analyses. The main findings of selected systematic reviews are summarized in Table A2 [12,13,14,34,35,36,37,38,39,40,41,42,43,44,45,46,47,48,49,50,51,52,53,54].

Thromboembolic complications in hospitalized COVID-19 patients range from 7.2 to 40.8% [39]. Regression models in one meta-analysis showed that increasing age was associated with a higher prevalence of VTE, DVT, and PE, while an increasing body mass index was associated with an increasing prevalence of PE [12]. Meta-analyses showed consistent results related to the high incidence of VTE in severely ill patients, particularly those admitted to the ICU. By contrast, a low risk of venous and arterial TE events was found in ambulatory and post-discharge COVID-19 patients; the risk is higher in post-discharge patients than in outpatients [53]. The proportions of VTE events in all patients, ambulatory, and post-discharge patients were 0.80% (95% CI, 0.44–1.28), 0.28% (95% CI: 0.07–0.64), and 1.16% (95% CI: 0.69–1.74), respectively.

Significant elevation of D-dimer (e.g., ≥3.00 µg/mL) has been associated with VTE development and can be used to identify high-risk patients [13,41,44,49,55]. Moreover, although infrequently reported, cerebral venous thrombosis has been found to occur in patients with COVID-19 infection [37].

Findings from a meta-analysis show a significant incidence of arterial thromboembolic events (acute myocardial infarction, acute ischemic stroke, and acute limb ischemia) in patients hospitalized for COVID-19 [53]. The pooled frequency of arterial TE events in this meta-analysis was 2.0% (95% PI, 0.4–9.6%). Compared to these findings, data from a large retrospective cohort study in non-hospitalized COVID-19 patients show lower rates of arterial TE events requiring hospitalization (1.01%; 95% CIs, 0.97% to 1.05% during the pre-vaccination period, and 1.06%; 95% CIs, 1.03% to 1.10% during the vaccination period), and in patients with influenza (0.45%; 95% CIs, 0.41% to 0.49%). The risk of arterial TE was higher for patients with COVID-19 during the pre-vaccination period (adjusted hazard ratio 1.53; 95% Cls, 1.38 to 1.69) and the vaccination period (1.69; 95% CIs, 1.53 to 1.86) than for patients with influenza [56]. Likewise, 90-day absolute risk of VTE with Covid-19 was 0.73% (0.70% to 0.77%) during the pre-vaccination period, 0.88% (0.84 to 0.91%) during the vaccination period, and 0.18% (0.16% to 0.21%) with influenza.

Similar findings were observed in the above-mentioned meta-analysis by Mansory et al. in ambulatory and post-discharge COVID-19 patients [53]. Arterial events occurred in 0.75% (95% CI: 0.27–1.47) of all patients, 1.45% (95% CI: 1.10–1.86) of post-discharge patients, and 0.23% (95% CI: 0.019–0.66) of ambulatory patients.

The high incidence of thrombotic complications observed in severely ill COVID-19 patients has drawn attention to the application of antithrombotic drugs in patients with COVID-19 infection. The thrombotic risk in hospitalized COVID-19 patients is elevated despite the use of anticoagulant prophylaxis. Results of a meta-analysis of 11 cohort studies (5 conducted exclusively in the ICU setting, and 6 in the ward setting, with 10% to 38% of patients requiring admission to the ICU) show that approximately 24% of patients developed VTE (12% PE and 12% DVT) despite anticoagulation with at least prophylactic dosing [41]. Patients in ICU had a higher risk of VTE (30.4%) than those in the ward (13.0%). For these reasons, the efficacy and safety of intermediate- or therapeutic-dose versus prophylactic-dose of anticoagulant in hospitalized COVID-19 patients is still object of clinical research.

A systematic review and meta-analysis of RCTs, in hospitalized COVID-19 patients of various severity, shows that therapeutic-dose anticoagulation is more effective in preventing thromboembolic events than prophylactic-dose, but significantly increases the risk of major bleeding [57]. Therefore, the risk–benefit ratio must be considered when choosing one of the two approaches.

Among noncritically ill patients hospitalized with COVID-19, the 30-day primary composite outcome was not significantly reduced with therapeutic-dose anticoagulation compared with prophylactic-dose anticoagulation. However, fewer patients who were treated with therapeutic-dose anticoagulation required intubation and fewer died [58]. Among patients with severe COVID-19 treated with corticosteroids and for whom pulmonary embolism was ruled out at hospital admission in most cases, the risks of mortality, thrombotic outcomes, and disseminated intravascular coagulation (DIC) were low at 30 days. Conclusions regarding the lack of benefit of anticoagulant therapy cannot be drawn because it is imprecise [59].

Of note, a large multicenter cohort study evaluated the incidence of TE complications and overall mortality in patients admitted to 8 hospitals in Netherlands during the first and second COVID-19 waves (between September and November 2020) [60]. Mortality was reduced by 47% in the second wave, but the thrombotic complication rate remained high and comparable to the first wave. Moreover, there is some evidence showing that the incidence and severity of PE did not significantly differ across the periods of ancestral strain and Alpha, Delta, and Omicron variants [61]. Despite the decreased pathogenicity of Omicron variants, the platelet phenotype closely resembles that observed in subjects infected with Delta, also in terms of thrombotic potential [62]. Hence, careful attention to provision of adequate thromboprophylaxis is invariably warranted.

## 4. Risk of VTE in Human Immunodeficiency Virus (HIV)-Infected Patients

Several studies have proven HIV infection to be a prothrombotic condition [16,63,64,65,66,67,68,69,70]. Available evidence shows that HIV-positive patients have several risk factors and a 2–10-fold increased risk of VTE compared to the general population [63]. A higher rate of VTE was observed in HIV-infected patients younger than 50 years of age (3.31% versus 0.53% in age-matched healthy controls, *p* < 0.0001), with a CD4+ cell count less than 200 cells/mm^3^ or with a diagnosis of acquired immunodeficiency syndrome (AIDS) [63,65,68]. In the general population the incidence rate of VTE is 1 to 2 per 1000 patient-years, but is highly age-dependent, ranging from 0.1 per 1000 patient-years under the age of 30 to 10 per 1000 patient-years in those over the age of 80. In a systematic review incidence rate of VTE per 1000 person-years among HIV-infected people was 2.8 (IQR: 2.5–3.0) [64].

Many factors are linked with VTE in patients with HIV, and these risk factors can be categorized in three groups: drugs risk factors, viral risk factors, and host risk factors, as summarized in Table 1 [70].

Despite the availability of highly active antiretroviral therapy (HAART), HIV-positive patients may be at higher risk of arterial vascular disease, possibly mediated by chronic inflammation, and consequently at higher risk of VTE. The risk of VTE will vary depending on traditional risk factors (e.g., inherited hypercoagulable states, hospitalization, surgery) and the severity of HIV (CD4 count, opportunistic infections, HIV-related malignancies). Due to an increased inflammatory state or the presence of concomitant comorbidities, advanced HIV infection is a risk factor for the development of thrombosis. HAART therapy does not appear to be significantly involved in the onset of VTE [70]. No associations between any specific antiretroviral drugs and risk of a venous thrombotic events have been found [71,72].

Reports indicate that HIV-infected patients have a higher risk of developing recurrent VTE compared to uninfected controls [67]. Pregnancy and the postpartum period are additional risk factors for VTE in HIV-infected women [70,73]. The risk in HIV-infected pregnant women is 120-fold higher than in HIV-positive controls, and 157-fold higher compared to HIV-negative pregnant women. Compared with HIV-negative controls, HIV-infected patients are also at increased risk of VTE after surgery [74].

A Danish nationwide population-based cohort study showed that HIV-infected patients are at increased risk of VTE, especially in the intravenous drug user population. HAART and possibly low CD4 cell count further increase the risk [68].

Thrombosis clinical appearance and distribution are like those observed in non-HIV individuals [64,70]. Patients with HIV should be observed and treated not only for opportunistic infections and HIV-related malignancies, but also for complications such as thrombosis [75]. TE events commonly occur in popliteal and femoral veins followed by PE. Patients with HIV who have unexplained chest pain, dyspnea, or hypoxemia should be investigated for PE.

VTE in HIV-infected patients should be managed as in non-HIV patients, including long-term prophylaxis with low molecular weight heparin and warfarin for patients with recurrent thrombosis. However, the interaction between antiretroviral agents and anticoagulants, particularly warfarin, needs to be considered [75,76,77,78]. Metabolic interaction between warfarin and antiretrovirals is likely, particularly if non-nucleoside reverse transcriptase inhibitors (NNRTIs) and protease inhibitors (PIs) are included in the antiretroviral regimen, given the influence of many antiretrovirals on CYP2C9.

## 5. Risk of VTE in Tuberculosis (TB)

Respiratory infections are well known to increase the risk of VTE complications, including DVT and PE [2]. It has been suggested that, in patients with TB, elevated plasma fibrinogen with reactive thrombocytosis results in the presence of a hypercoagulable state [79,80]. As demonstrated in several acute infections, these changes likely result from the inflammatory cascade triggered by chronic infection [81,82]. The results of a meta-analysis of 9 cross-sectional studies and case series (16,190 patients) show that VTE is not rare among patients with active TB. The prevalence of VTE was 3.5% (95% CI 2.2–5.2), with a prevalence of pulmonary PE of 5.8% (95% CI 2.2–10.7) and of DVT of 1.3% (95% CI 0.8–2.0) [83]. Patients with active tuberculosis had a higher risk of VTE (OR 2.90; 95% CI 2.30–3.67), DVT (OR 1.56; 95% CI 1.14–2.14), and PE (OR 3.58; 95% CI 2.54–5.05) compared to those without TB.

Data from a very large US database, adjusted for many classic VTE risk factors, suggest that TB is an independent risk factor for VTE [84]. In-hospital mortality of patients with both active TB and VTE [11/72 (15%)] was significantly higher than mortality of patients with only active TB [92/3413 (2.7%)] or only VTE [5062/199,480 (2.5%)]. Moreover, PE was more frequent in black patients, suggesting that this population, which is also more likely to suffer from TB, should be followed carefully.

Effective directly observed treatment short-course (DOTS) with anti-TB drugs helps reduce the probability of VTE [85]. Anticoagulation with vitamin K antagonists in patients receiving anti-TB treatment is often complicated by drug interactions, especially with rifampicin [86]. Further studies are needed to establish the safety of novel oral anticoagulants (NOAs) in this clinical scenario. For now, a viable alternative to warfarin may be represented by low-molecular-weight heparin (LMWH).

## 6. Risk of VTE in HCV-Infected Patients

The occurrence of VTE in people with HCV infection has been the object of several retrospective cohort and cross-sectional studies. Two systematic reviews with meta-analysis are available on this topic [17,87]. In the largest of these reviews, Ambrosino et al. included 6 studies (5 retrospective cohorts, 1 cross-sectional), for a total of 100,364 HCV patients as compared with 8,471,176 uninfected controls [17]. The review showed a significantly increased of VTE risk (OR, 1.9; 95% CI: 1.4–2.5; *p* < 0.0001) and DVT risk (OR: 1.9; 95%CI: 1.3–2.7; *p* < 0.0001), but not of PE (4 studies, OR: 1.8; 95% CI: 0.8–3.6; *p* = 0.09). The increased VTE risk was confirmed in the analysis of four studies reporting adjusted risk estimates (OR: 1.87; 95%CI: 1.32–2.65; *p* = 0.0001), and after excluding studies with populations exposed to transient risk factors for VTE (4 studies, OR: 1.49; 95% CI: 1.16–1.91; *p* = 0.001).

These data suggest that HCV infection alone, in the absence of cirrhosis, is associated with an increased risk of thromboembolic events. VTE, as a consequence of HCV infection, has several causes: vasculopathy caused by chronic inflammation, presence of anticardiolipin and antiphospholipid antibodies, downregulation of anticoagulant proteins and upregulation of procoagulant proteins, higher rates of thrombin generation in patients with HCV and cirrhosis, and higher cryoglobulinemia in patients with HCV which can cause thrombotic vasculitis [70,88,89].

## 7. Risk of VTE in CMV-Infected Patients

In the published literature there is increasing evidence that human CMV may play a role in thrombotic disorders [18,90]. It is hypothesized that CMV may reside in both superficial and deep arteries and veins, triggering endothelial and vascular inflammation that increases the risk of thrombus formation; currently, the most accepted theory as the major mechanism for thrombosis formation in CMV infection is the transient hypercoagulable state due to the production of antiphospholipid syndrome (APS)-associated and anticardiolipin antibodies [91,92]. Acute CMV infection has been associated with an increased short-term (up to 6 months from the index date) VTE risk [18]. The incidence rates per 1000 capita of VTE among CMV-IgM seropositive and CMV-IgM seronegative patients were 3.06 (19 patients) and 1.36 (115 patients), respectively (OR, 2.25; 95% CI, 1.38–3.66; *p* = 0.003). CMV-IgM seropositivity was independently associated with VTE appearance (OR 2.49; 95% CI 1.53–4.06; *p* < 0.0001) following adjustment for age, sex, and other confounders. CMV-IgM seropositivity was not associated with arterial thrombosis. DVT/PE, splanchnic vein thrombosis, and splenic infarction were the most prevalent thromboses associated with acute CMV infection.

CMV infection has been increasingly recognized as a thrombotic risk factor in immunocompetent patients but remains a possible contributing factor of VTE in immunocompromised patients, such as HIV-infected and renal transplant patients [16,75,93]. Coagulation disorders and estroprogestative contraception must be taken into a great deal of consideration in patients with CMV infection, since they could be important risk factors for VTE [94].

The role of CMV in vasculopathy and VTE has been underestimated because the infection, in immunocompetent adults, is predominantly subclinical. Prompt administration of anticoagulants is beneficial in patients with CMV-related thrombosis.

## 8. Risk of VTE in Patients with Other Infections

A relationship between typhoid fever and thrombosis has been known for over a century [95]. Venous thrombosis was a common complication of typhoid fever before the antibiotic era and often occurred in the fourth week of illness. However, after the introduction of effective antimicrobials, thrombosis and phlebitis occur rarely. Of note, more recently in a nationwide population-based cohort study, non-typhoidal salmonellosis was associated with an increased risk of new-onset DVT and PE [96].

Risk of VTE in patients with inflammatory bowel disease (IBD) is well established, and patients with IBD are at increased risk of developing *Clostridium difficile* infection [97,98]. Rate of VTE was higher in hospitalized patients with IBD with *C. difficile* compared with those without it [99]. Hospitalized patients with *C. difficile* infection carry an almost 2-fold higher risk of DVT and 1.5-fold higher risk of PE compared to those without *C. difficile* after adjusting for potential confounding factors [99,100]. This increased risk of TE events merits consideration of DVT prophylaxis in hospitalized patients with *C. difficile* and warrants further study.

Other bacterial agents that have been associated with an increased risk of VTE are *Chlamydia pneumoniae*, *Mycoplasma pneumoniae*, and *Orientia tsutsugamushi* (the aetiologic agent of scrub typhus) [101,102,103,104,105]. During bacterial infections, the production of reactive oxygen species contributes to platelet activation and stimulation of the coagulation cascade.

Among other viral agents that have been associated with increased risk of VTE should be mentioned other herpes virus (e.g., Epstein-Barr Virus (EBV), Varicella-Zoster Virus (VZV), Herpes Simplex Virus (HSV) [106,107,108,109,110]). Although there is insufficient evidence to support a correlation between Zika virus (ZIKV) or Chikungunya virus (CHIKV) infection and VTE, the presence of elevated D-dimer levels would indicate that ZIKV or CHIKV infection may contribute to an increased risk of VTE [111]. On the other hand, a significantly increased risk of VTE was found in the period following hemorrhagic fever with renal syndrome (HFRS), a mild viral hemorrhagic fever caused by Puumala hantavirus [112]. The major concern with dengue fever is the hemorrhagic event, related to thrombocytopenia, dysfunctional platelets, coagulation pathway abnormalities, and prolonged shock [113]. However, even if not frequently, TE complications have been described during dengue acute infection [114,115]. The likeliness of VTE occurrence related to dengue is linear with the severity of the dengue infection and the patient’s length of stay in hospitals.

Some VTE events have been described in the literature as a complication in patients with influenza; the H1N1 strain may be more involved in thromboembolic events [116].

## 9. Thromboprophylaxis of Infection-Related VTE

Extended duration (for up to 45 days) VTE prophylaxis may not be warranted for all infections but may be very helpful for some [117]. The association between infection and increased risk of VTE, after adjustment for known risk factors, has been demonstrated in previous studies; the highest risk is reported in subjects with intra-abdominal infection, followed by systemic bloodstream infection, lower respiratory tract infection, such as pneumonia, and symptomatic urinary tract infection [19,118,119]. Although patients with sepsis will generally receive low-molecular-weight heparin to prevent DVT/PE, there are no current antithrombotic treatment schemes used specifically for patients with infection [10]. Prophylaxis with rivaroxaban reduced the occurrence of VTE among patients hospitalized with acute infectious diseases after adjustment for known risk factors [117].

For hospitalized COVID-19 patients of various severity, when compared to a lower-dose regimen, higher-dose anticoagulants result in little to no difference in all-cause mortality and increased minor bleeding up to 30 days [120].

The Padua prediction score is a tool used to assess hospitalized medical patients at risk of VTE, including those with infectious diseases [121].

In HIV-infected people, the potential for dangerously interactions between anticoagulants and antiretroviral agents should be always considered. Low-molecular-weight heparin (LMWH) should be considered in some cases in the HIV-infected population receiving HAART, given the absence of compliance in some HIV-affected subjects. LMWH should be a safer choice compared to warfarin in those patients, always keeping in mind that HIV infection may be an independent risk factor for the development of heparin-induced thrombocytopenia [122].

## 10. Discussion

VTE, encompassing both DVT and PE, remains an important cause of morbidity and mortality. Various factors increase the risk of VTE, including prolonged immobilization, surgery, cancer, spine injuries, limb fractures, and infections. Infections can significantly increase the risk of developing VTE. Both acute and chronic infections are characterized by immune dysregulation that interacts with the coagulation cascade; the inflammatory state is an important trigger for the activation of coagulation factors.

In this review, we have summarized the available evidence related to the epidemiology of VTE caused by infections, in patients with infection in both community settings and in hospitals. Hospitalization with infection is a strong VTE trigger also in non-immobilized patients. Many types of infections can elevate the risk of VTE, although some appear to increase risk more than others. It is well recognized that patients with SARS-CoV-2, HIV, tuberculosis, HCV, and CMV infections are at increased risk of VTE.

Results from case–control studies show that, after adjusting for other common VTE risk factors, any infection (pneumonia, urinary tract infections, intrabdominal infections, blood stream infections), both in hospital and community settings, increased significantly the probability of VTE.

Moreover, we tried to evaluate how the risk of VTE in person with different types of infections could be addressed in clinical practice with the use of anticoagulants. Extended-duration VTE prophylaxis may not be warranted for all infections, but may be very helpful for some, such as those with intra-abdominal infection, systemic bloodstream infection, lower respiratory infection, and symptomatic urinary tract infection. Currently, there are no known antithrombotic treatment regimens used specifically for patients with infection, although patients with sepsis are commonly given low-molecular-weight heparin for prevention of DVT/PE.

In COVID-19 patients, a therapeutic dose of anticoagulant can be considered in both non-severe and severe cases to reduce thrombosis. Therapeutic-dose anticoagulation is more effective in preventing thromboembolic events than prophylactic-dose but may be associated with increased bleeding events.

The management of VTE in HIV-infected patients and in patients with TB should be the same as for other patients, but the interaction between antiretroviral agents (such as NNRTIs and PIs), rifampin, and anticoagulants (e.g., warfarin) needs to be considered.

## 11. Conclusions

In this narrative review we provide up-to-date and comprehensive information on VTE and infectious disease, and we also tried to evaluate how the risk of VTE in person with different types of infections could be addressed in clinical practice. Infection significantly increases the risk of venous thrombosis and/or pulmonary embolism, that are contributors to both morbidity and mortality. Our review underlines the fact that infection needs to be considered as an important cause of VTE, particularly in hospitalized patients, and that awareness of signs and symptoms of VTE, particularly of PE, is especially important.

Narrative reviews have inherent limitations in terms of objectivity, completeness of literature search, and interpretation of findings, and the current review is not exempt of these limitations. Nonetheless, we had the possibility to analyze a wide variety of studies, providing an overall summary of an important and timely topic, requiring further attention and knowledge.

## Figures and Tables

**Table 1:** Risk factors (RF) for VTE in HIV-infected people. Adapted from Zerangian et al. [70].

Viral RF	Drugs RF	Host RF
CD4 cells count	Megestrol acetate	Age
IRIS	HAART	Hypercoagulable state
Opportunistic infections: - CMV - *Mycobacterium tuberculosis* - *Mycobacterium avium*-intracellulare - Pneumocystosis		- Deficiency of protein B - Deficiency of protein C - Deficiency of antithrombin
HIV viral load		Endothelial dysfunction
HIV-associated malignancies		Miscellaneous factors of haemostasis
		Antiphospholipid antibodies
		- Von Willebrand factor - Tissue factors - Microbial translocation

IRIS: immune reconstitution inflammatory syndrome; HAART: highly active antiretroviral therapy (HAART); CMV: citomegalovirus.

## Appendix A

**Table A1:** Thromboembolism and infectious diseases. Main findings from population-based cohort studies, nationwide databases, and RCTs.

1st Author, Year Ref	Aim	Design	Population	Main Results	Notes
Cohoon, 2018 [19]	To evaluate the occurrence of infection (overall and specific infection) in people with deep vein thrombosis and pulmonary embolism.	Population-based Case control study.	Pts with deep vein thrombosis and/or pulmonary embolism over 35-year period in a US population of 144,248 individuals.	513/1303 (39.4%) cases and 189/1494 (12.7%) controls had an infection in the previous 92 days. Unadjusted OR = 4.5; 95% CI: 3.6–5.5 (*p* < 0.0001).	In a multivariate analysis adjusting for other common venous thromboembolism risk factors, any infection (pneumonia, UTI, intrabdominal infections, blood stream infections) increased the odds of venous thromboembolism (OR 2.4; 95% CI: 1.8–3.2; *p* < 0.0001).
Alikhan, 2004 [2]	To evaluate different types of acute medical illness and predefined factors (chronic heart and respiratory failure, age, previous VTE, and cancer) as risk factors for VTE.	Logistic regression analysis from a RCT.	Hospitalized pts with acute medical illness (heart failure, respiratory failure, infection, rheumatic disorder, inflammatory bowel disease).	The primary univariate analysis showed that the presence of an acute infectious disease, age older than 75 years, cancer, and a history of VTE were statistically significantly associated with an increased VTE risk. Multiple logistic regression analysis indicated that these factors were independently associated with VTE.	Multivariate analysis shows that acute infection was an independent risk factor for VTE: OR, 1.74 (95% CIs, 1.12–2.75), *p* = 0.02.
Smeeth, 2006 [8]	To investigate whether acute infections increase the risk of VTE.	Records from general practices who had registered pts with the UK’s Health Improvement Network database between 1987 and 2004.	A self-controlled case-series method to study the risk of first DVT (n = 7278) and first PE (n = 3755) after acute respiratory and UTI.	The risks of DVT and PE were significantly raised, and were highest in the first two weeks, after UTI. The incidence ratio for DVT was 2.10 (95% CI 1.56–2.82), and that for PE 2.11 (1.38–3.23). The risk gradually fell over the subsequent months, returning to the baseline value after 1 year. The risk of DVT was also higher after respiratory tract infection.	Acute infections are associated with a transient increased risk of VTE events in a community setting. Our results confirm that infection should be added to the list of precipitants for VTE and suggest a causal relation.
Levine, 2008 [22]	Venous and arterial TE in severe sepsis.	A retrospective analysis of data from 3 RCTs.	A total of 2649 pts in ICU with known or suspected infection and sepsis-associated acute organ dysfunction.	84 of 2649 pts (3.2%; 95% Cl, 2.5–3.9%) developed at least one TE event over 28 days. Nearly 3/4 of episodes were atheroembolic (n = 62); 25% involved the deep venous system (n = 25). Ischemic stroke (n = 30) and venous TE (n = 25) each occurred in about 1% of pts.	Clinically manifest TE occurred in about 3% of severe sepsis pts treated in the ICU. Arterial TE may be more common than previously recognized.
Clayton, 2010 [23]	Recent respiratory infections and risk of TE.	Case–control study through a general practice database.	All cases aged ≥18 years of first-time diagnosis of DVT or PE were identified together with single-matched controls from a primary care general practice database.	There were 457/11,557 (4.0%) DVT cases with respiratory infection in the year before the index date (73 in the preceding month) compared with 262/11,557 (2.3%) controls (24 in the preceding month). There was an increased risk of DVT in the month following infection [adjusted odds ratio (OR) = 2.64, 95% confidence interval (95% CI) 1.62–4.29] which persisted up to a year.	There are strong associations between recent respiratory infection and VTE.
Rothberg, 2011 [3]	Risk factor model to predict venous TE in hospitalized medical pts.	Retrospective cohort study.	Pts admitted to 374 US hospitals with a primary diagnosis of pneumonia, heart failure, COPD, stroke, and UTI.	Of 242,738 pts, 612 (0.25%) pts fulfilled our criteria for VTE during hospitalization, and an additional 440 (0.18%) were readmitted for VTE within 30 days (overall incidence of 0.43%). In the multivariable model, age, sex, and additional risk factors were associated with VTE. The strongest risk factors were inherited thrombophilia (OR 4.00), length of stay ≥6 days (OR 3.22), inflammatory bowel disease (OR 3.11), central venous catheter (OR 1.87), and cancer.	The risk of symptomatic VTE in general medical pts is low.
Schmidt, 2011 [24]	To evaluate whether hospital-diagnosed infections or infections treated in the community increase the risk of VTE.	Population-based case– control study in northern Denmark using medical databases.	We identified all pts with a first hospital-diagnosed VTE during the period 1999–2009 (n = 15,009). For each case, we selected 10 controls from the general population matched for age, gender, and county of residence (n = 150,074). We used the regional prescription database to identify all antibiotic prescriptions filled by cases and controls 1 year before their index date.	Respiratory tract, urinary tract, skin, intra-abdominal, and bacteraemic infections diagnosed in hospital or treated in the community were associated with a greater than equal to twofold increased VTE risk: 3.3 (95% CI: 2.9–3.8) and 2.6 (95% CI: 2.5–2.8), respectively.	Compared with individuals without infection during the year before VTE, the IRR for VTE within the first 3 months after infection was 12.5 (95% CIs: 11.3–13.9) for pts with hospital-diagnosed infection and 4.0 (95% CI: 3.8–4.1) for pts treated with antibiotics in the community. Adjustment for VTE risk factors reduced these IRRs to 3.3 (95% CI: 2.9–3.8) and 2.6 (95% CI: 2.5–2.8), respectively.
Del Principe, 2013 [25]	To determine: (1) the risk factors associated with CVC-related thrombosis (CRT) and their frequency in a homogeneous population of pts with AML; (2) the impact of an antithrombotic prophylaxis using LMWH on CRT occurrence.	Retrospectively cohort of 71 consecutive AML pts.	Hospitalized pts with acute myeloid leukemia (AML).	Occurrence of CRT was significantly associated with CVC-exit site infections (14/19, *p* = 0.01) and sepsis (16/19, *p* = 0.005) with no difference between LMWH and no-LMWH group.	In multivariate analysis, both CVC-exit site infections and sepsis were confirmed to be independent risk factors for CRT development.
Dalager-Pederson, 2014 [9]	To evaluate the risk of VTE within one year of CAB in comparison to that in matched controls.	Danish cohort study. A regression analyses with adjustment for confounding factors was used to compare the risk of VTE in bacteraemia pts and controls.	4213 adult CAB pts who had positive blood cultures drawn on the day of hospital admission, 20,084 matched hospitalised controls admitted for other acute medical illness, and 41,121 matched controls from the general population.	Among CAB pts, 1.1% experienced VTE within 90 days of admission and 0.5% during 91–365 days after admission. The adjusted 90-day odds ratio (OR) for VTE was 1.9 (95% CI 1.4–2.7) compared with hospitalised controls, and 23.4 (95% CI 12.9–42.6) compared with population controls.	The risk of VTE is substantially increased within 90 days after community-acquired bacteraemia when compared to hospitalised controls and population controls. However, the absolute risk of VTE following CAB is low.
Barr, 2014 [26]	To establish risk of VTE in pts treated for bacterial infection in the community with outpatient parenteral antimicrobial therapy.	Retrospective cohort.	Out-pts with bacterial infection.	VTE incidence of 2/780 (0.26%, 95% CI: 0.03–0.92%).	The study found a low incidence of VTE in OPAT pts and does not support routine application of inpatient VTE prophylaxis algorithms to pts treated for infection in the community.
Frasson, 2015 [27]	RIETE Investigators. Infection as cause of immobility and occurrence of VTE analysis of 1635 medical cases from a registry.	Data were collected from the worldwide RIETE registry (47,390 pts), including pts with symptomatic objectively confirmed VTE and followed-up for at least 3 months.	Subgroup of non-surgical pts with infection leading to immobility and reported. As risk factor/cause for VTE by the attending physicians. Pts with infection were compared to those with dementia causing immobility (dementia, differently from infection, is not known as relevant risk factor for VTE).	Compared with pts immobilized due to dementia, pts with infection had a shorter duration of immobilization prior to VTE (less than 4 weeks in 94.2 vs. 25.9% of cases). During the 3-month follow-up, VTE pts with infection versus those with dementia had a lower rate of fatal bleeding (0.5 vs. 1.1%; *p* < 0.05) or fatal PE (1.7 vs. 3.5%; *p* < 0.01). Pts with respiratory tract infections had more likely PE as initial VTE presentation than other types of infection (62.3 vs. 37.7%; *p* < 0.001). Significantly more pts with pneumonia than those with other respiratory infections had received VTE prophylaxis (50.2 vs. 30.6%; *p* < 0.001).	Infection seems to contribute to the pathogenesis of VTE by accelerating the effects of immobility.
Cowan, 2017 [28]	To explore the relationship between hospitalization with infection and short-term VTE risk.	A case-crossover design and conditional logistic regression were used.	Hospitalized infections among VTE cases with corresponding control periods 1 year and 2 years prior. Data collected from prospective ARIC cohort.	Of the 845 total VTE cases, 75 had a hospitalization with an infection in the 90 days preceding the VTE event.	Hospitalized infection is a trigger of VTE. There is an association between hospitalization with infection and subsequent short-term VTE risk that exceeds the known association between hospitalization and VTE.
Grimnes, 2017 [20]	To investigate the impact of hospitalization with acute infection on the VTE-risk in pts with and without concomitant immobilization, and to explore the differential impact RTI and UTI tract infections on the risk of DVT and PE.	A population-based crossover study. Hospitalizations and VTE-triggers were registered during the 90 days before a VTE (hazard period) and in four preceding 90-day control periods.	VTE-pts (n = 707) recruited from a general population.	Acute infection was registered in 267 (37.8%) of the hazard periods and in 107 (3.8%) of the control periods, corresponding to a high VTE-risk after infection (OR 24.2, 95% CI 17.2–34.0), which was attenuated to a 15-fold increased after adjustment for immobilization. The risk was 20-fold increased after infection without concomitant immobilization, 73-fold increased after immobilization without infection, and 141-fold increased with the two combined.	Hospitalization with infection is a strong VTE trigger also in non-immobilized pts. Infection and immobilization had a synergistic effect on the VTE-risk.
Carpenter, 2019 [29]	NS results in hypercoagulability and increased risk of infection. Furthermore, infection increases the risk of VTE. Our objective was to determine the prevalence of infection, VTE, and the associated outcomes among a cohort of hospitalized children with NS.	Retrospective cohort.	A cohort of hospitalized children with NS.	730 hospitalizations occurred among 370 children with NS. 148 children (40%) had ≥1 infection (211 episodes) and 11 (3%) had VTE.	Hospitalized children with NS have high rates of infection. Presence of VTE was associated with infection. Both were associated with longer hospitalizations and ICU stays.
Eck, 2021 [30]	ICU pts	A pooled analysis of two prospective cohort studies.	2208 pts admitted to ICU.	The prevalence of any VTE during 3 months before ICU admission was 3.6%. Out of 2166 pts, in ICU, 47 (2.2%; 95% CI 1.6–2.9%) developed PE-LDVT and 38 pts (1.8%; 95% CI 1.2–2.4%) developed NLDVT. Renal replacement therapy (OR 3.5: 95% CI 1.4–8.6), respiratory failure (OR 2.0; 95% CI 1.1–3.8), and previous VTE (OR 3.6; 95% CI 1.7–7.7) were associated with PE-LDVT. Central venous catheters (OR 5.4; 95% CI 1.7–17.8) and infection (OR 2.2; 95% CI 1.1–4.3) were associated with NLDVT.	Thrombotic events are common in critically ill pts, both before and after ICU admittance. Development of PE-LDVT but not NLDVT was associated with increased mortality.
Smilowitz, 2021 [31]	Risk of thrombotic events after respiratory infection requiring hospitalization.	US nationwide database 2012-14. Pts admitted with asthma or cellulitis served as comparators. Readmissions for acute myocardial infarction (MI) and VTE were evaluated at 30 to 180 days.	Pts discharged after a respiratory infection.	Among 5,271,068 pts, 0.56% and 0.78% were readmitted within 30-days with MI and VTE, respectively. Relative to asthma and cellulitis, respiratory infection was associated with a greater age and sex-adjusted hazard of 30-day readmission for MI (adjusted HR [aHR] 1.48 [95% CI 1.42–1.54] vs. asthma; aHR 1.36 [95% CI 1.31–1.41] vs. cellulitis) and VTE (aHR 1.28 [95% CI 1.24–1.33] vs. asthma; aHR 1.26, [95% CI 1.22–1.30] vs. cellulitis).	Hospitalization for respiratory infection was associated with increased risks of thrombosis that were highest in the first 30-days after discharge and declined over time.
Angriman, 2022 [3]	To determine whether surviving a first sepsis hospitalization is associated with long-term cardiovascular events.	Population-based matched cohort study.	Septic adult pts survivors of a first sepsis hospitalization were matched to adult survivors of a non-sepsis hospitalization using hard-matching and propensity score methods.	Sepsis survivors experienced an increased hazard of major cardiovascular events compared to non-sepsis survivors (HR 1.30; 95% CI 1.27–1.32), which was more pronounced in younger (<40 yrs) pts (HR 1.66; 95% CI 1.36–2.02).	Sepsis survivors also faced an increased hazard of VTE (HR 1.61; 95% CI 1.55–1.67) and all-cause death (HR 1.26; 95% CI 1.25–1.27).
Pisani, 2022 [32]	To evaluate VTE risk in the pre-COVID-19 era in a large ICU database.	A database for ICU pts.	Consecutive pneumonia pts admitted to the ICU.	The 30-day cumulative incidence of VTE was 7%. Mortality was 20.6% among pts with VTE and 19.2% among those without VTE.	VTE risk in ICU pts with pneumonia was high and decreased with thromboprophylaxis. The diagnosis of VTE did not substantially affect the risk of death.

VTE: venous thromboembolism; RCT: randomized controlled trial; DVT: deep venous thrombosis; PE: pulmonary embolism; UTI: urinary tract infections; ICU: intensive care unit; COPD: chronic obstructive pulmonary disease; CVC: central venous catheters; AML: acute myeloid leukemia; LMWH: low-molecular-weight-heparin; CAB: community acquired bacteraemia; OPAT: outpatient parenteral antimicrobial therapy; ARIC: atherosclerosis risk in communities; RTI: respiratory tract infections; NS: nephrotic syndrome; NLDVT: non-lower deep vein thrombosis; LDVT: lower extremity deep venous thrombosis; MI: myocardial infarction.

**Table A2:** Thromboembolic complications in COVID-19 patients. Main findings of selected systematic reviews.

1st Author, Year Ref	Clinical Setting	No. of Studies	Outcomes	Results	Main Conclusions
Birkeland, 2020 [34]	Hospitalized pts.	14 observational studies (1677 pts).	VTE incidence; d-dimer level.	VTE incidence was 26.9% (95% CI 20.8–33.1). D-dimer was higher for the VTE cohort (5.62 [SD 0.9] vs. 1.43 [SD 0.6]; *p* < 0.001.	Despite the utilization of background anticoagulation, VTE incidence was high. Odds of VTE were higher in the ICU (OR 6.38, 95% CI 3.67–11.11; *p* < 0.001) but lower with anticoagulation (OR 0.58, 95% CI 0.36–0.92; *p* = 0.02).
Di Minno, 2020 [12]	Hospitalized pts with different severity of COVID-19 (from 0 to 100% ICU admission).	20 observational studies (1988 pts).	VTE, DVT, PE incidence.	Mean prevalence (MP) of VTE was 31.3% (95% CI: 24.3–39.2%); MP of DVT was 19.8% (95% CI: 10.5–34.0%); MP of PE was 18.9% (95% CI: 14.4–24.3%).	The rate of TE complications in COVID-19 pts is definitely high, particularly in pts admitted to ICUs. Rate of TE complications remain high also among pts under antithrombotic prophylaxis.
Lu, 2020 [35]	Hospitalised COVID-19 pts with various severity of infection	25 observational studies (20 on VTE incidence and 5 on the relationship between anticoagulation and mortality).	To determine the incidence of VTE and evaluate the role of anticoagulation in pts with COVID-19.	Pooled incidence rates of VTE, PE, and DVT were 21% (95% CI 15–27%), 15% (95% CI 10–20%), and 27% (95% CI 19–36%), respectively.	The incidence of VTE among hospitalised COVID-19 pts was high. Rates of VTE were higher among pts admitted to the ICU; antithrombotic therapy was not associated with a lower mortality risk (RR = 0.86, 95% CI, 0.69–1.09).
Porfidia, 2020 [36]	Hospitalized pts with COVID-19.	30 observational studies (3487 pts).	Incidence of VTE in pts with COVID-19.	Incidence of VTE was 26% (95% PI, 6–66%). PE with or without DVT occurred in 12% of pts (95% PI, 2–46%) and DVT alone in 14% (95% PI, 1–75%). In pts admitted to ICU, VTE occurred in 24% (95% PI, 5–66%), PE in 19% (95% PI, 6–47%), and DVT alone in 7% (95% PI, 0–69%). Corresponding values in general wards were respectively 9% (95% PI, 0–94%), 4% (95% PI, 0–100%), and 7% (95% CI, 1–49%).	VTE represents a frequent complication in hospitalized COVID-19 pts and often occurs as PE.
Tu, 2020 [37]	COVID-19 pts with CVT.	9 studies and 14 COVID-19 pts with CVT were studied.	Incidence of CVT.	A significant proportion of pts had raised D-dimer (75.0%) and CRP levels (50.0%). Two pts reported presence of antiphospholipid antibodies. Most pts received anticoagulation (91.7%) while overall mortality rate was 45.5%.	Whilst infrequently reported, CVT has been found to occur in pts with COVID-19 infection. The unusually high mortality rate warrants a high index of suspicion from physicians, and early treatment with anticoagulation should be initiated in these settings.
Zhang, 2020 [38]	Hospitalized COVID-19 pts.	17 retrospective cohort studies (1913 pts).	VTE (DVT, PE) occurrence.	The pooled incidence of VTE was 25% (95% CI, 19–31%), with a significant difference between the incidence of PE (19%; 95% CI, 13–25%) and DVT (7%; 95% CI, 4–10%).	VTE incidence was 25% in hospitalized COVID-19 pts. Higher incidence of VTE was observed in COVID-19 pts with a severe condition or with a low rate of pharmacologic thromboprophylaxis.
Kunutsor, 2020 [39]	Hospitalised pts.	35 observational cohort studies (9249 pts).	VTE, PE, DVT.	The pooled incidence was 18.4% (12.0–25.7) for VTE (n = 19 studies), 13.5% (8.4–19.5) for PE (n = 22 studies) and 11.8% (7.1–17.4) for DVT (n = 18 studies).	There is a high incidence of TE complications in hospitalized COVID-19 pts (from 7.2 to 40.8%), which appears to be driven by VTE disease. These TE complications are remarkably high in COVID-19 infection despite the use of thromboprophylaxis. The most frequently diagnosed venous TE complication in the overall population is PE; the incidence of TE complications is substantially higher in severe COVID-19 disease compared to the overall population, with a higher incidence of DVT than PE.
Sridharan, 2020 [40]	Hospitalized pts. with different disease severity.	11 observational studies (1478 pts).	VTE events.	Pooled rate of major VTE was 12.5% in hospitalized pts. and 17.2% in ICU pts. When therapeutic anticoagulation dosing was compared with prophylactic anticoagulation, the pooled OR of VTE was 0.33 (95% CIs 0.14–0.75; *p* = 0.008) suggesting statistical significance with therapeutic dosing of anticoagulation for primary prevention of VTE in all hospitalized pts.	Major VTE events, especially pulmonary embolism, seem to be high in COVID-19 pts admitted to the ICU. Therapeutic anticoagulation dosing seems to significantly benefit the odds of preventing any VTE when compared with prophylactic dosing in all hospitalized pts.
Chi, 2020 [41]	Preliminary evidence indicates that prophylactic-dose thromboprophylaxis may be inadequate to control the increased risk of VTE in pts hospitalized for COVID-19.	11 cohort studies.	Frequency of VTE and death among COVID-19 pts who received thromboprophylaxis on hospitalization. The endpoints included VTE, PE, DVT, and mortality.	Among hospitalized COVID-19 pts, 23.9% (95% CI, 16.2% to 33.7%;) developed VTE despite anticoagulation. PE and DVT were detected in 11.6% (95% CI, 7.5% to 17.5%) and 11.9% (95% CI, 6.3% to 21.3%;) of pts, respectively. Pts in the ICU had a higher risk of VTE (30.4%; 95% CI, 19.6% to 43.9%) than those in the ward (13.0%; 95% CI, 5.9% to 26.3%). The mortality was estimated at 21.3% (95% CI, 17.0% to 26.4%).	COVID-19 pts who developed VTE had higher D-dimer levels than those who did not develop VTE (mean difference, 2.05 µg/mL; 95% CI, 0.30 to 3.80 µg/mL; *p* = 0.02). Prominent elevation of D-dimer may be associated with VTE development and can be used to identify high-risk subset.
Hasan, 2020 [42]	COVID-19 admitted to ICU.	12 studies.	VTE.	The pooled prevalence of VTE among ICU pts receiving prophylactic or therapeutic anticoagulation across all studies was 31% (95% CI 20–43%). Subgroup pooled analysis limited to studies reporting prophylactic anticoagulation alone and mixed (therapeutic and prophylactic anticoagulation) reported pooled prevalences of VTE of 38% (95% CI 10–70%) and 27% (95% CI 17–40%), respectively.	With a high prevalence of thromboprophylaxis failure among COVID-19 pts admitted to ICU, individualised rather than protocolised VTE thromboprophylaxis would appear prudent at interim.
Srivastava, 2021 [43]	COVID-19 pts	13 cohort studies (6648 pts).	Association between VTE and diseases severity.	Pts with PE and DVT are at increased risk of being admitted to ICU (RR: 2.21; 95% CI: 1.86–2.61; *p* < 0.001) and (RR: 2.69; 95% CI: 2.37–3.06; *p* < 0.001), respectively.	This study highlights the need to consider measures for reducing thromboembolism risk amongst COVID-19 pts.
Gabbai-Armelin, 2021 [13]	Hospitalized pts with different severity of COVID-19.	20 studies (case–control, cohort) were included in the qualitative analysis, and 6 in the meta-analysis.	Prognostic factors for TE.	Hypertension and diabetes were the comorbidities more frequently associated with thrombolytic events. Significant results were found regarding D-dimer (*p* < 0.0001) and age (*p* = 0.0202) for TE.	Pts older than 60 years, with hypertension, diabetes, and D-dimer values above 3.17 µg/mL can be considered prognostic factors for developing TE due to COVID-19.
Liu, 2021 [44]	Hospitalized pts.	26 studies (18 retrospective, 6 prospective observational, and 2 cross-sectional) for a total of 4382 pts.	Incidence of VTE, DVT, PE.	The total incidence of VTE was 28.3% (95% CI, 21.6–35.4%), with an incidence of 38.0% (95% CI, 29.1–47.4%) and 17.2% (95% CI, 11.4–23.8%) among those with severe and general COVID-19, respectively. The total incidence of DVT of the lower extremities was 18.3% (95% CI, 10.8–27.2%), ranging from 22.1% (95% CI, 11.0–35.5%) and 12.8% (95% CI, 5.0–23.3%) in those with severe and general COVID-19, respectively. The total incidence of PE was 17.6% (95% CI, 12.3–23.5%), with a rate of 21.7% (95% CI, 14.8–29.3%) in severe cases and 12.5% (95% CI, 6.1–23.5%) in general cases.	The occurrence of VTE, DVT, and PE has been substantial among hospitalized pts with COVID-19, especially among those with severe COVID-19. Pts with severe COVID-19 and VTE had significantly greater mortality compared with similar pts without VTE. An increased D-dimer level might be an indicator of the occurrence of VTE in pts with COVID-19.
Mai, 2021 [45]	Hospitalized pts with or without COVID-19. With similar disease severity. Within each study, groups were comparable in terms of ICU admission rates, sex, age, and methods for VTE diagnosis.	7 observational studies (41,768 pts).	To compare the rate of VTE between COVID-19 and non-COVID-19 cohorts with similar disease severity.	The overall risk of VTE (RR 1.18; 95%CI 0.79–1.77; *p* = 0.42), PE (RR 1.25; 95% CI 0.77–2.03; *p* = 0.36; I2 = 52%) and deep venous thrombosis (RR 0.92; 95%CI 0.52–1.65; *p* = 0.78) did not significantly differ between COVID-19 and non-COVID-19 cohorts. However, subgroup analyses suggested an increased risk of VTE amongst COVID-19 versus non COVID-19 cohorts when only pts hospitalized within the ICU were considered (RR 3.10; 95% CI 1.54–6.23), which was not observed in cohorts of predominantly non-ICU pts (RR 0.95; 95%CI 0.81–1.11).	There was no signal for a difference in VTE in COVID-19 cohorts compared to non-COVID-19 cohorts, except for the subgroup of pts hospitalized in the ICU.
Mansory, 2021 [46]	COVID-19 pts hospitalized in ICU or in non-ICU wards.	91 observational cohort studies (35,017 pts). 35 studies included ICU pts only, 23 studies had non-ICU pts only, and 33 studies included both ICU and non-ICU pts.	To evaluate the epidemiology of VTE, DVT, PE in hospitalized ICU and non-ICU pts.	The overall frequency of VTE in all pts, ICU and non-ICU, was 12.8% (95% CI: 11.10–14.60), 24.1% (95% CI: 20.07–28.28), and 7.7% (95% CI: 5.95–9.70), respectively. PE occurred in 8.5% (95% CI: 6.91–10.20), and proximal DVT occurred in 8.2% (95% CI: 6.67–9.87) of all hospitalized pts. The relative risk of VTE associated with ICU admission was 2.99 (95% CI: 2.301–3.887, *p* < 0.001).	This study confirmed a high risk of VTE in hospitalized COVID-19 pts, especially those admitted to the ICU. Nevertheless, sensitivity analysis suggests that previously reported frequencies of VTE in COVID-19 might have been overestimated.
Tufano, 2021 [47]	COVID-19 and other pulmonary infection cohorts, in particular H1N1, and in an ICU setting.	12 cohort studies (prospective and retrospective, +1 autoptic study) (1,013,495 pts).	To compare the occurrence of VTE, PE, and DVT between COVID-19 and other pulmonary infection cohorts, in particular H1N1, and in an ICU setting.	We observed a high RD between COVID-19 and non-COVID-19 pts for VTE (6% more risk as compared with non-COVID-19) and PE, in particular in pts admitted to the ICU. (11% more risk in ICU)	It is not completely clear why some infections such as COVID-19 have a strong influence on coagulation and are associated with thrombosis, while in others this effect is limited.
Wu, 2021 [48]	Critically ill COVID-19 pts.	19 observational studies with 1599 pts.	Prevalence of VTE, DVT, and PE.	Prevalence of VTE, DVT, and PE was 28.4% [95% CI: 20.0–36.8%], 25.6% (95% CI: 17.8–33.4%), and 16.4% (95% CI: 10.1–22.7%), respectively. Limited to studies, in which all pts received routine prophylactic anticoagulation, the prevalence for VTE, DVT, and PE was 30.1% (95% CI: 19.4–40.8%), 27.2% (95% CI: 16.5–37.9%), and 18.3% (95% CI: 9.8–26.7%), respectively.	Critically ill pts with COVID-19 have a high prevalence of VTE, despite the use of present routine prophylactic anticoagulation.
Kollias, 2021 [49]	Hospitalized pts with different severity of illness.	17 studies (3973 pts.) reported PE, and 32 studies (2552 pts.) reported DVT.	Prevalence of VTE, DVT, and PE.	Pooled PE prevalence of 32% (95% CI: 25, 40%) and DVT prevalence of 27% (95% CI: 21, 34%) were reported.	Meta-regression analysis showed that the prevalence of VTE was higher across studies with a higher percentage of ICU pts and higher study population mean D-dimer values. Hospitalized pts with severe COVID-19 are at high VTE risk despite prophylactic anticoagulation.
Gallastegui, 2021 [50]	Hospitalized pts with different severity of illness.	57 studies.	Incidence of PE.	PE incidence among all hospitalized COVID19 pts was 7.1% (95% CI: 5.2%, 9.1%). Pts admitted to ICUs had higher PE incidence (13.7%; 95% CI: 8.0%, 20.6%).	PE incidences among hospitalized COVID19 pts are much lower than has been previously postulated based on smaller, often biased study reports.
Jenner, 2021 [51]	ICU-treated pts with COVID-19.	28 studies (2928 pts).	Prevalence of VTE, DVT, and PE.	Thrombotic complications occurred in 34% of ICU-managed pts, with DVT reported in 16.1% and PE in 12.6% of pts.	Studies adopting systematic screening for venous thrombosis with duplex ultrasound reported a significantly higher incidence of DVT compared to those relying on clinical suspicion (56.3% vs. 11.0%, *p* < 0.001).
Zuin, 2021 [52]	COVID-19 pts after hospital discharge.	11 studies (18,949 pts).	Incidence of VTE (DVT, PE).	The cumulative post-discharge rate of VTE in COVID-19 pts ranged between 0.2 and 14.8%. The pooled incidence is 1.8% (95% CI: 0.8–4.1).	Meta-regression analysis revealed that the post-discharge incidence of VTE events was directly affected by age (*p* = 0.03) and male gender (*p* = 0.04) and inversely correlated with the length of follow-up period (*p* = 0.012). Conversely, no associations were identified using postdischarge thromboprophylaxis (*p* = 0.20), cancer (*p* = 0.14), VTE history (*p* = 0.82), ICU admission (*p* = 0.55), and mean length of hospitalization (*p* = 0.68).
Lobbes, 2021 [14]	COVID-19 in ICU.	21 studies (5296 pts).	To evaluate risk factors for VTE.	A moderate-certainty evidence was found for an association between VTE and the D-dimer peak (OR 5.83, 95% CI: 3.18–10.70), and length of hospitalization (OR 7.09, 95% CI: 3.41–14.73) and intubation (OR 2.61, 95% CI: 1.94–3.51), and a low-certainty evidence for an association between VTE and CRP (OR 1.83, 95% CI: 1.32–2.53), D-dimer (OR 4.58, 95% CI: 2.52–8.50), troponin T (OR 8.64, 95% CI: 3.25–22.97), and the requirement for inotropic drugs (OR 1.67, 95% CI: 1.15–2.43).	Traditional VTE risk factors (i.e., history of cancer, previous VTE events, obesity) were not found to be associated to VTE in COVID-19. Anticoagulation was not associated with a decreased VTE risk. VTE RF in severe COVID-19 correspond to individual illness severity and to inflammatory and coagulation parameters.
Mansory, 2022 [53]	In ambulatory and discharged COVID-19 pts.	16 cohort studies (11 retrospective and 5 prospective). 102,779 pts.	To evaluate the epidemiology of VTE and arterial thrombosis events in ambulatory and postdischarge COVID-19 pts.	The overall proportion of VTE events in all pts (ambulatory and post-discharge) was 0.80% (95% CI: 0.44–1.28), 0.28% (95% CI: 0.07–0.64), and 1.16% (95% CI: 0.69–1.74), respectively. Arterial events occurred in 0.75% (95% CI: 0.27–1.47) of all pts, 1.45% (95% CI: 1.10–1.86) of post-discharge pts, and 0.23% (95% CI: 0.019–0.66) of ambulatory pts.	This study found a low risk of venous and arterial thrombi in ambulatory and postdischarge COVID-19 pts, with a higher risk in post-discharge pts compared with ambulatory pts. This suggests that regular universal thromboprophylaxis in these patient populations is probably not necessary.
Candeloro, 2023 [54]	Hospitalized pts with COVID-19.	36 trials (28 retrospective cohorts, 5 prospective cohorts, and 3 RCTs), 100,949 pts.	To evaluate the frequency of ATEs, AMI, AIS, ALI, or other ATE.	The pooled ATE frequency was 2.0% (95% PI, 0.4–9.6%). The pooled ATE frequency for AMI, AIS, ALI, and other ATE was 0.8% (95% PI, 0.1–8.1%), 0.9% (95% PI, 0.3–2.9%), 0.2% (95% PI, 0.0–4.2%), and 0.5% (95% PI, 0.1–3.0%), respectively.	There was a non-negligible proportion of ATE in pts. hospitalized for COVID-19. The results are similar to those found in hospitalized pts with influenza or with non-COVID viral pneumonia.
Dutch COVID and Thrombosis Coalition, 2021 [60]	947 pts hospitalized in 8 Dutch hospitals during the first and second COVID-19 waves.	1 multicenter cohort study.	Incidence of thrombotic complications and overall mortality in COVID-19 pts admitted to 8 Dutch hospitals between 1 September and 30 November 2020.	144 pts died (15%). The adjusted cumulative incidence of all thrombotic complications after 10, 20 and 30 days was 12% (95% CI: 9.8–15%), 16% (13–19%) and 21% (17–25%), respectively. Pts characteristics between the first and second wave were comparable. The adjusted HR for overall mortality in the second wave versus the first wave was 0.53 (95% CI: 0.41–0.70). The adjusted HR for any thrombotic complication in the second versus the first wave was 0.89 (95% CI: 0.65–1.2).	Mortality was reduced by 47% in the second wave, but the thrombotic complication rate remained high, and comparable to the first wave. Careful attention to provision of adequate thromboprophylaxis is invariably warranted.

VTE: venous thromboembolism; ICU: intensive care unit; DVT: deep venous thrombosis; PE: pulmonary embolism; MP: mean prevalence; TE: thromboembolic; CVT: cerebral venous thrombosis; CRP: C-reactive protein; ATE: arterial thromboembolic events; AMI: acute myocardial infarction; AIS: acute ischemic stroke; ALI: acute limb ischemia; HR: hazard ratio.
